# Prospective Analysis Between Neutrophil-to-Lymphocyte Ratio on Admission and Development of Delirium Among Older Hospitalized Patients With COVID-19

**DOI:** 10.3389/fnagi.2021.764334

**Published:** 2021-11-23

**Authors:** Eduardo Fernández-Jiménez, Ainoa Muñoz-Sanjose, Roberto Mediavilla, Gonzalo Martínez-Alés, Iker I. Louzao, Jorge Andreo, Susana Cebolla, María-Fe Bravo-Ortiz, Carmen Bayón

**Affiliations:** ^1^Department of Psychiatry, Clinical Psychology and Mental Health, La Paz University Hospital, Madrid, Spain; ^2^Hospital La Paz Institute for Health Research (IdiPAZ), Madrid, Spain; ^3^Department of Psychiatry, Universidad Autónoma de Madrid (UAM), Madrid, Spain; ^4^Centro de Investigación Biomédica en Red (CIBERSAM), Madrid, Spain; ^5^Department of Epidemiology, Columbia University Mailman School of Public Health, New York, NY, United States

**Keywords:** neutrophil-to-lymphocyte ratio (NLR), delirium, COVID-19, hospitalization, older adults

## Abstract

**Objective:** To examine any prospective association between neutrophil-to-lymphocyte ratio (NLR) at hospital admission and subsequent delirium in older COVID-19 hospitalized patients comparing by sex and age groups.

**Methods:** The sample consisted of 1,785 COVID-19 adult inpatients (minimum sample size required of 635 participants) admitted to a public general hospital in Madrid (Spain) between March 16th and April 15th, 2020. Variables were obtained from electronic health records. Binary logistic regression models were performed between baseline NLR and delirium adjusting for age, sex, medical comorbidity, current illness severity, serious mental illness history and use of chloroquine and dexamethasone. An NLR cut-off was identified, and stratified analyses were performed by age and sex. Also, another biomarker was tested as an exposure (the systemic immune-inflammation index –SII).

**Results:** 55.3% of the patients were men, with a mean age of 66.8 years. Roughly 13% of the patients had delirium during hospitalization. NLR on admission predicted subsequent delirium development (adjusted OR = 1.02, 95 percent CI: 1.00–1.04, *p* = 0.024). Patients between 69 and 80 years with NLR values > 6.3 presented a twofold increased risk for delirium (*p* = 0.004). There were no sex differences in the association between baseline NLR and delirium (*p* > 0.05) nor SII predicted delirium development (*p* = 0.341).

**Conclusion:** NLR is a good predictor of delirium during hospitalization, especially among older adults, independently of medical comorbidity, illness severity, and other covariates. Routine blood tests on admission might provide valuable information to guide the decision-making process to be followed with these especially vulnerable patients.

## Introduction

Delirium is an acute neuropsychiatric disorder with a fluctuating course that impairs consciousness, attention, and cognitive function (Inouye et al., 2014) and affects around 1 in 4 hospitalized patients (American Psychiatric Association [APA], 2013). Older adult patients among 62–82 years experiencing delirium during the intensive care unit (ICU) stay are at increased risk for cognitive dysfunction after hospital discharge (Pandharipande et al., 2013; Müller et al., 2020).

One out of three hospitalized COVID-19 patients endure neuropsychiatric manifestations (e.g., headache, paresthesia, or disturbed consciousness) during hospitalization (Nalleballe et al., 2020) which is associated with disease severity (Mao et al., 2020): two thirds of COVID-19 patients admitted to intensive care units (ICUs) experience agitation and half are diagnosed with delirium (Helms et al., 2020a). Moreover, among the most severe cases with COVID-19, delirium is often the only sign of the infection (Beach et al., 2020; O’Hanlon and Inouye, 2020). As with most reasons for acute-care hospital admission, especially among older adults (Witlox et al., 2010), delirium is a strong predictor of mortality in hospitalized COVID-19 patients (Maguire et al., 2021).

Determinants of delirium among COVID-19 patients include systemic inflammation and neuroinflammation (following the cytokine storm driven by SARS-CoV-2 infection) (Fajgenbaum and June, 2020; Pensato et al., 2021; Perrin et al., 2021), organ dysfunction (e.g., respiratory or kidney failure), thrombosis, use of deep sedative strategies, prolonged mechanical ventilation, isolation (Ellul et al., 2020; Helms et al., 2020b; Kotfis et al., 2020) and specific medications for COVID-19 (Hamm and Rosenthal, 2020; Wu et al., 2021).

The role of delirium as a predictor of morbidity and mortality among hospitalized patients, including patients admitted due to COVID-19, calls for cost-effective detection strategies. Neutrophil-to-lymphocyte ratio (NLR) is an indicator of the immune-inflammatory response available from routine blood tests performed at hospital admission. NLR is a good predictor of poor prognosis of COVID-19 (Yang et al., 2020; Kerboua, 2021; Pandurangan et al., 2021); a non-specific transdiagnostic marker for neuropsychiatric disorders (Mazza et al., 2018; Brinn and Stone, 2020) including delirium (Egberts and Mattace-Raso, 2017; Oh et al., 2017; Zhao et al., 2021); and a prognosis marker in cardiovascular disease (Wang et al., 2014), kidney disease (Solak et al., 2013), and sepsis (Huang et al., 2020). To our knowledge, no study has examined the link between NLR and delirium in COVID-19 hospitalized patients. This study aimed to explore any prospective association between NLR measured at hospital admission and subsequent development of delirium during hospitalization among COVID-19 patients.

## Materials and Methods

### Participants

We used the electronic health records of La Paz University Hospital, a large teaching hospital that provides care to a catchment area of roughly 527,000 people in Madrid (Spain). During the study period, hospitals could not admit patients from outside their catchment area (Martínez-Alés et al., 2021). We included all patients aged 16 years and over admitted with a COVID-19 diagnosis for at least 24 h between March 16th and April 15th, 2020 (*n* = 1,785). According to methods to estimate minimum sample size required for multiple logistic regression analyses (Peduzzi et al., 1996) with 8 predictors and 12.6% patients with the outcome (delirium), this study would need a sample size greater than 635 participants. The study period included both the day with the highest number of confirmed COVID-19 cases (3,218 on March 20th) and the day with the highest number of confirmed deaths (702 on March 27th) during the initial pandemic outbreak in Madrid (Institute of Health Carlos, 2020). The study was approved by the Hospital La Paz Ethics Committee (Borobia et al., 2020) and was conducted in accordance with the Helsinki Declaration, as revised in 1993.

### Measures

- Pre-hospitalization variables: age, sex, serious mental illness history (as a proxy of previous use of antipsychotics) and comorbidity burden as measured by the Charlson Comorbidity Index (CCI) (Charlson et al., 1987).

- Baseline variables, registered at hospital presentation: neutrophil-to-lymphocyte ratio (NLR) and the systemic immune-inflammation index –SII– (platelets x neutrophils/lymphocytes).

- Variables registered during hospitalization: (1) delirium –diagnosed by psychiatrists from the Liaison Psychiatry Department based on repeated clinical examinations using DSM-5 diagnostic criteria (American Psychiatric Association [APA], 2013); (2) critical illness –defined as being admitted to an ICU or having two or more recorded blood oxygen saturation measurements less than 90% while on a reservoir mask or on non-invasive mechanical ventilation (Martínez-Alés et al., 2021); (3) use of chloroquine (yes/no) and/or dexamethasone (yes/no); and (4) all-cause in-hospital mortality.

### Data Analysis

First, we summarized the characteristics of the sample. Second, we analyzed the association between our exposure of interest, baseline NLR (continuous variable), and subsequent development of delirium (yes/no), using binary logistic regression models and reporting odds ratios (ORs) with 95 percent confidence intervals (95 percent CI). Receiver Operating Characteristic (ROC) curves were calculated to stablish an optimal NLR cut-off value for the outcome, and additional binary logistic regression models were conducted using the resulting dichotomized NLR variable as exposure. All models were adjusted for the following predictors of the outcome based on prior knowledge and existing evidence (Wang et al., 2020; Mychajliw et al., 2021): age, sex, CCI, current illness severity, and two COVID-19 medications (chloroquine and/or dexamethasone) during hospitalization. We then conducted stratified analyses based on age and sex. In a set of sensitivity analyses, we included serious mental illness history as an additional covariate, included a different biomarker as an exposure (the systemic immune-inflammation index) and removed strong and influential outliers. Absence of multicollinearity and linearity of the exposure and its log odds assumptions were verified. All statistical analyses were conducted with IBM SPSS Statistics 28.0.

## Results

### Descriptive Analyses

Our sample consisted of 986 males (55.3%), with a mean (standard deviation) age of 66.8 (16.6) (see Table 1). The mean (SD) NLR on admission was 7.2 (7.1). Delirium was diagnosed in 225 patients (12.7%).

**TABLE 1:** Characteristics of the participants.

		Age subgroups			
	All (*n* = 1,785)	16–55 (*n* = 412)	56–68 (*n* = 445)	69–80 (*n* = 458)	81–99 (*n* = 470)
Sex [female] (%)	798 (44.7)	202 (49)	176 (39.6)	194 (42.4)	226 (48.1)
Delirium (%)	225 (12.7)	7 (1.7)	15 (3.4)	59 (12.9)	144 (30.6)
Critical patient (%)	146 (8.2)	26 (6.3)	36 (8.1)	46 (10.0)	38 (8.1)
Death (%)	394 (22.3)	12 (3.0)	37 (8.4)	119 (26.2)	226 (48.5)
Age, *M* (*SD*)	66.8 (16.6)	43.4 (8.9)	61.0 (3.6)	73.7 (3.4)	86.0 (4.2)
CCI, *M* (*SD*)	3.5 (2.6)	0.7 (1.0)	2.4 (1.6)	4.4 (1.9)	6.0 (1.9)
NLR, *M* (*SD*)	7.2 (7.1)	5.3 (4.0)	6.8 (7.3)	8.1 (8.0)	8.4 (7.7)

*CCI, Charlson Comorbidity Index (range = 0–14); NLR, neutrophil-to-lymphocyte ratio. M, mean; SD, standard deviation.*

### Primary Analyses

Our results showed a direct association between baseline NLR and subsequent development of delirium during hospitalization (adjusted OR = 1.02, 95 percent CI: 1.00–1.04; *B* = 0.021; *S.E.* = 0.009; *p* = 0.024) after controlling for age, sex, CCI, current illness severity, chloroquine, and/or dexamethasone.

ROC curve analyses set the cut-off value of NLR at 6.3 (sensitivity = 51% and specificity = 64%, with AUC = 0.60). NLR levels higher than 6.3 were associated, in adjusted regression models, with a 1.37 times increased probability of delirium (95 percent CI: 1.00–1.88; *B* = 0.315; *S.E.* = 0.160; *p* = 0.049) (see Table 2).

**TABLE 2:** Prediction of NLR on delirium development adjusting for covariates.

							95% C.I. for EXP(B)	
	*B*	*S.E.*	Wald	*df*	*p*	Exp(B)	Lower	Upper
Constant	–8.089	0.705	131.484	1	<0.001	0.00		
Sex	–0.109	0.164	0.442	1	0.506	0.90	0.65	1.24
Age	0.076	0.009	70.813	1	<0.001	1.08	1.06	1.10
CCI	0.088	0.041	4.653	1	0.031	1.09	1.01	1.18
Current illness severity	0.449	0.271	2.754	1	0.097	1.57	0.92	2.66
Chloroquine	–0.016	0.328	0.002	1	0.961	0.98	0.52	1.87
Dexamethasone	0.020	0.312	0.004	1	0.949	1.02	0.55	1.88
NLR	0.315	0.160	3.870	1	0.049	1.37	1.00	1.88

*CCI, Charlson Comorbidity Index; NLR, neutrophil-to-lymphocyte ratio.*

### Stratified Analyses

Age was divided into four subgroups based on quartiles (≤55 years; > 55 and ≤ 68; > 68 and ≤ 80; and > 80 years). Of note, among patients aged > 68 and ≤ 80 years, baseline NLR levels higher than the 6.3 cutoff were associated, in adjusted regression models (including sex, CCI, current illness severity as covariates), with a 2.34 (95 percent CI: 1.30–4.19, *p* = 0.004) times increased probability of delirium. Odds ratios for the remaining age subgroups were as follows: among ≤ 55 years, OR = 0.35 (95 percent CI: 0.04–3.12, *p* = 0.344); > 55 and ≤ 68 years, OR = 1.19 (95 percent CI: 0.38–3.73, *p* = 0.762); and > 80 years, OR = 1.16 (95 percent CI: 0.77–1.74, *p* = 0.470). No differences in terms of sex were found in the association between baseline NLR and risk of delirium (males: adjusted OR = 1.28, 95 percent CI: 0.84–1.94, *p* = 0.254; and females: adjusted OR = 1.54, 95 percent CI: 0.96–2.45, *p* = 0.074) after controlling for age, CCI and current illness severity.

### Sensitivity Analyses

An additional analysis was conducted to control for the influence of previous use of antipsychotics including as a proxy the serious mental illness history, along with the remaining covariates (age, sex, CCI, current illness severity, chloroquine, and/or dexamethasone). In this new model, odds ratio for subsequent development of delirium based on an NLR value > 6.3 at hospital admission did not change (OR = 1.36, 95 percent CI: 0.99–1.87; *B* = 0.309; *S.E.* = 0.162; *p* = 0.057) regarding the aforementioned analyses.

Moreover, a new model with another biomarker as an exposure (the systemic immune-inflammation index –SII) showed no association between SII levels on admission and subsequent delirium development (adjusted OR = 1.00, 95 percent CI: 1.00–1.00; *B* = 0.000; *S.E.* = 0.000; *p* = 0.341) after adjusting for all covariates (age, sex, CCI, current illness severity, serious mental illness history, chloroquine, and/or dexamethasone).

Finally, after removal of outliers –defined as individuals with baseline NLR values over 40 points, based on previous studies (Yang et al., 2020), the adjusted association between baseline NLR levels higher than 6.3 and subsequent delirium remained unchanged (OR = 1.33, 95 percent CI: 0.97–1.84; *B* = 0.286; *S.E.* = 0.164; *p* = 0.082) after adjusting for all the covariates (age, sex, CCI, current illness severity, serious mental illness history, chloroquine, and/or dexamethasone).

## Discussion

This is the first study to explore any prospective association between NLR levels at hospital admission and subsequent development of delirium during hospitalization among COVID-19 patients focusing especially on older adults. We found that high NLR increased the risk of delirium during hospitalization in predictive models adjusted for age, sex, medical comorbidity, illness severity, and use of chloroquine and dexamethasone. Further, we found that a cut-off NLR value of 6.3 serves to differentiate patients at markedly higher risk of delirium, especially among patients aged > 68 and ≤ 80 years. Our results should provide clinicians with a readily applicable, relatively unexpensive tool to support clinical assessments of risk of delirium among older adult hospitalized patients with COVID-19. Also, these findings should provide valuable information for decision-makers, who shall guide the implementation of evidence-based protocols adapted to critical illness patients with systemic injury (Kotfis et al., 2020).

These findings add to the body of evidence supporting use of biomarkers (Androsova et al., 2015), such as C-reactive protein (Zhang et al., 2014; Dillon et al., 2017), tumor necrosis factor (Kazmierski et al., 2014), and other interleukins (Westhoff et al., 2013) to predict risk of delirium during hospital admission in acutely ill patients. Of note, increased levels of NLR have been reported to have higher predictive value in delirium than CRP, neutrophils and lymphocytes (Egberts and Mattace-Raso, 2017). Another systemic inflammation biomarker (SII) –which considers together neutrophil, platelet, and lymphocyte– did not predict delirium development in this study, in spite of the relationship between an increase of platelets levels and delirium (Oyama et al., 2021). Notably, NLR levels are less costly to obtain than cellular mediators and acute phase reactants (Zhao et al., 2021) and neutrophil and lymphocyte counts are part of basic routine determinations in most patients considered for hospital admission due to medical conditions. In addition, our findings expand the increasing use of NLR levels to classify patients’ risk of a variety of outcomes, including risk of depression following hemorrhagic stroke and risk of all-cause mortality in hospitalized COVID-19 patients (Gong et al., 2020; Haghjooy Javanmard et al., 2020).

Importantly, we found that baseline NLR levels predict risk of subsequent development of delirium in models adjusted for other relevant clinical predictors of delirium onset. For instance, critical patients are at an increased risk of delirium –largely due to the effects of anesthetics, immobilization, isolation, and life-support measures– especially prolonged mechanical ventilation (Illendula et al., 2020; Kotfis et al., 2020; Parker et al., 2020; Bellelli et al., 2021; Hawkins et al., 2021). Also, chloroquine and dexamethasone, which were often used to treat hospitalized COVID-19 patients during the initial pandemic outbreak in spring 2020, seemingly increase risk of delirium (Hamm and Rosenthal, 2020; Wu et al., 2021), especially when used in combination (Doyno et al., 2021). While these factors should continue to be taken into account in clinical assessments of risk of delirium, our results suggest that NLR levels may serve as an independent risk indicator for patients across levels of medical comorbidity, disease severity, and types of drugs used.

Over one in ten patients in our sample presented delirium during hospitalization, diagnosed by means of clinical assessments by trained psychiatrists, in line with most recent American Psychiatric Association’s estimates (12–24%) among hospitalized patients (American Psychiatric Association [APA], 2013). Risk of delirium among hospitalized COVID-19 patients increases exponentially with disease severity: around 50% of critical COVID-19 patients develop delirium during admission (Helms et al., 2020b; Pun et al., 2021). Because delirium is a major predictor of mortality among hospitalized COVID-19 patients (Maldonado, 2018), early detection efforts to identify COVID-19 patients at high risk of delirium are a major unmet clinical need. In this sense, identifying a good predictor for delirium, like NLR, may enhance the treatment of causes for this condition, beyond the SARS-CoV-2 infection, other medical comorbidities, social isolation, etc.

Our study has limitations. First, we did not include additional important covariates in our models, such as type of respiratory support, cognitive impairment measures (Eschweiler et al., 2021; Mychajliw et al., 2021) or use of other medications, due to limited data availability. We adjusted for the use of chloroquine, dexamethasone and for presence of critical illness, defined as being admitted to an ICU or having two or more recorded blood oxygen saturation measurements less than 90% while on a reservoir mask or on non-invasive mechanical ventilation, which is a proxy of the use of sedative and analgesic agents. Second, as a single-center study, transportability of our results to other contexts may be somewhat limited. Notably, Spain’s tax-funded National Health System provides universal coverage to the entire population with a focus on equitable access to care. In addition, as mentioned, hospitals did not admit patients from outside their catchment areas during the initial pandemic outbreak in Madrid. Accordingly, we have no reason to believe that important selection processes may have introduced substantial selection bias in our estimates, which can be considered roughly population-based (see Supplementary Table 1). Likewise, this study has several strengths. First, the prospective nature of our study design let temporal precedence between the exposure (NLR) and the outcome (delirium). Also, our study is based on real world data from clinical practice. Last, La Paz University Hospital was an important COVID-19 hotspot in the world during the first outbreak of the pandemic.

In conclusion, delirium was common among patients hospitalized due to COVID-19 in our study. We found that elevated NLR on admission was independently associated with increased risk of delirium in COVID-19 inpatients –most notably among patients aged > 68 and ≤ 80 years. This study provides evidence supporting use of baseline NLR levels as a component of clinical assessments for delirium risk among hospitalized COVID-19 patients, and potentially among patients admitted due to other acute conditions. Clinicians should monitor blood-test NLR levels as an early detection effort to identify older adult patients at risk of delirium, in order to provide them with evidence-based prevention strategies in a current context where this population, early vaccinated this year, could be losing protection against the new coronavirus variants.

## Data Availability Statement

The raw data supporting the conclusions of this article will be made available by the authors, without undue reservation.

## Ethics Statement

The studies involving human participants were reviewed and approved by Comité de Ética para la Investigación del Hospital Universitario La Paz. Written informed consent for participation was not required for this study in accordance with the national legislation and the institutional requirements.

## Author Contributions

EF-J, RM, AM-S, GM-A, IL, JA, SC, M-FB-O, and CB conceptualized and designed the investigation. EF-J analyzed the data. RM and GM-A verified the statistical analysis. EF-J, RM, AM-S, and CB wrote the first version of the manuscript with input from GM-A. RM, GM-A, and JA contributed to data collection and management. All authors provided valuable feedback, reviewed, edited and approved the final version of the manuscript.

## Conflict of Interest

The authors declare that the research was conducted in the absence of any commercial or financial relationships that could be construed as a potential conflict of interest.

## Publisher’s Note

All claims expressed in this article are solely those of the authors and do not necessarily represent those of their affiliated organizations, or those of the publisher, the editors and the reviewers. Any product that may be evaluated in this article, or claim that may be made by its manufacturer, is not guaranteed or endorsed by the publisher.
