# Supplementary material for: Prospective Analysis Between Neutrophil-to-Lymphocyte Ratio on Admission and Development of Delirium Among Older Hospitalized Patients With COVID-19
Source: Front Aging Neurosci. 2021 Nov 23;13:764334. doi: 10.3389/fnagi.2021.764334 (PMC8650500; doi:10.3389/fnagi.2021.764334)
Supplement: Supplementary file 1 [file Table_1.DOCX]

Supplementary Material

| Supplementary Table 1. Selected area-level socioeconomic and public health indicators for the catchment area under study. | | | | | |
| --- | --- | --- | --- | --- | --- |
|  |  |  | **Northern districts ^a^** | | |
|  | **Madrid** |  | **Tetuán** | **Fuencarral** | **All** |
| Population | 3,182,175 |  | 153,789 | 238,756 | 392,545 |
| Mean age (years) | 44 |  | 44 | 43 | 43 |
| Immigrant (%) | 8.4 |  | 13.7 | 4.7 | 8.2 |
| Adults unemployed (%) | 8.7 |  | 8.6 | 6.7 | 7.4 |
| Adults with bachelor´s degree (%) | 19.1 |  | 18.3 | 18.8 | 18.6 |
| Average annual income per person (€) | 21,146 |  | 20,629 | 22,469 | 21,748 |
| Houses in good repair (%) | 92.8 |  | 90.1 | 96.2 | 93.8 |
| Adults with chronic illness (%) | 69.7 |  | 72.7 | 70.1 | 71.1 |
| Adults with disability (%) | 5.6 |  | 5.2 | 4.4 | 4.7 |
| Source: Board of Indicators of Madrid´s Districts and Neighborhoods 2017: Sociodemographic Study of Districts. Accessed 2 Oct 2020. Available from <https://datos.madrid.es/portal/site/egob>.  ^a^ The Northern Catchment Area is composed by two districts: Tetuán and Fuencarral. | | | | | |
